# Supplementary material for: Dynamic transcriptomic profiles of zebrafish gills in response to zinc supplementation
Source: BMC Genomics. 2010 Oct 11;11:553. doi: 10.1186/1471-2164-11-553 (PMC3091702; doi:10.1186/1471-2164-11-553)
Supplement: Additional file 2 — Interactive Direct Interaction Network representing the molecular interactions between zinc, copper, iron, calcium and proteins encoded by transcripts changed by zinc supplementation. Mini web-site containing index.html and hyperlinked pages in subdirectory describing a Direct Interaction Network automatically generated based on curated interactions contained within the proprietary PathwayArchitect database. Ovals represent proteins and the circles symbolize metal ions. Objects are coloured by their abundance in zebrafish at the time-point they were significantly different from the control is a scale from -4 fold (dark green) to +4 fold (dark red). Where significant differences were found at more than one time-point, the colour overlay shows expression at the first instance. Dark blue squares denote 'binding', and light blue squares 'expression'; green squares stand for 'regulation', green diamonds for 'metabolism', and green circles for 'promoter binding'. Arrow heads indicate directionality of the interaction where annotated. All nodes and edges can be further interrogated by selecting the relative area of the image. [file 1471-2164-11-553-S2.zip › PathwayArchitect Zn xs DIN/1427910.html]

# REGULATION:

|  |  |
| --- | --- |
| Type | REGULATION |
| Effect | Negative |


---

|  |  |
| --- | --- |
| Score | 0 |


---

|  |  |
| --- | --- |
| Reference Count | 6 |


---

|  |  |
| --- | --- |
| Mechanism | Unknown |


---

|  |  |
| --- | --- |
| Reference:0 || Sentence | "Zinc pretreatment has been shown in vitro (rat myoblasts) to induce metallothionein (MT) and inhibit cadmium (Cd)-induced protooncogenes c-myc and c-jun mRNA levels. therefore, the purpose of this study was to determine whether the mRNA expression of the protooncogene c-jun as well as the tumor suppressor gene p53 is increased by Cd in the intact animal and, more specifically, in the target organ for Cd toxicity, the liver." |
| PMID | 8619230 |
| Year | 1996 |
| Species | Mouse |
| Journal | Toxicol Appl Pharmacol |
| RefScore | 2 |
| Source | PArchNLP |
  |
|  |  |


---

|  |  |
| --- | --- |
 Reference:1 || Sentence | Consistent with this, LA and NAC prevented zinc deficiency-induced activation of the early steps of NF- kappaB (IkappaBalpha phosphorylation) and AP-1 [c-Jun-N-terminal kinase (JNK) and p38 phophorylation] cascades, and the high NF-kappaB- and AP-1-DNA binding activities in total cell extracts. |
| Year | 2006 |
| PMID | 16298762 |
| Species | Human |
| Journal | Free Radic Res |
| RefScore | 1 |
| Source | PArchNLP |
  ||  |  |


---

|  |  |
| --- | --- |
 Reference:2 || Sentence | Inhibition of transcriptional activities of AP-1 and c-Jun by a new zinc finger protein ZNF394. |
| Year | 2004 |
| PMID | 15249231 |
| Species | Human |
| Journal | Biochem Biophys Res Commun |
| RefScore | 2 |
| Source | PArchNLP |
  ||  |  |


---

|  |  |
| --- | --- |
 Reference:3 || Sentence | A novel human KRAB-containing zinc-finger gene ZNF446 inhibits transcriptional activities of SRE and AP-1. |
| PMID | 15936718 |
| Year | 2005 |
| Species | Human |
| Journal | Biochem Biophys Res Commun |
| RefScore | 2 |
| Source | PArchNLP |
  ||  |  |


---

|  |  |
| --- | --- |
 Reference:4 || Sentence | ZNF328, a novel human zinc-finger protein, suppresses transcriptional activities of SRE and AP-1. |
| PMID | 15964554 |
| Year | 2005 |
| Species | Human |
| Journal | Biochem Biophys Res Commun |
| RefScore | 2 |
| Source | PArchNLP |
  ||  |  |


---

|  |  |
| --- | --- |
 Reference:5 || Sentence | ZNF325, a novel human zinc finger protein with a RBaK-like RB-binding domain, inhibits AP-1- and SRE-mediated transcriptional activity. |
| PMID | 16806083 |
| Year | 2006 |
| Species | Human |
| Journal | Biochem Biophys Res Commun |
| RefScore | 0 |
| Source | PArchNLP |
  |


---

|  |  |
| --- | --- |
